# Replicating state Quitline innovations to increase reach: findings from three states

**DOI:** 10.1186/s12889-019-8104-3

**Published:** 2020-01-06

**Authors:** Paula A. Keller, Rebecca K. Lien, Laura A. Beebe, Jane Parker, Paola Klein, Randi B. Lachter, Stephen Gillaspy

**Affiliations:** 1 ClearWay Minnesota SM, 8011 34th Ave S, Suite 400, Minneapolis, MN 55425 USA; 2 219 Main St. SE, Suite 302, Minneapolis, MN 55414 USA; 3 Hudson College of Public Health, The University of Oklahoma Health Sciences Center, 801 NE 13th St, Room 317, Post Office Box 26901, Oklahoma City, OK 73126-0901 USA; 4 Florida Department of Health, 4052 Bald Cypress Way, Tallahassee, FL 32399 USA; 5 Oklahoma Tobacco Research Center, 655 Research Pkwy #400, Oklahoma City, OK 73104 USA; 6 The University of Oklahoma College of Medicine, Stanton L Young Blvd, Oklahoma City, OK 73117 USA

**Keywords:** Quitline, Evaluation, Tobacco cessation, Smoking, Nicotine replacement therapy, Public health

## Abstract

**Background:**

Reaching tobacco users is a persistent challenge for quitlines. In 2014, ClearWay Minnesota^SM^ changed its quitline services and media campaign, and observed substantial increases in reach and strong quit outcomes. Oklahoma and Florida implemented the same changes in 2015 and 2016. We examined whether the strategies used in Minnesota could be replicated with similar results.

**Methods:**

We conducted a cross-sectional observational study of Minnesota’s QUITPLAN® Services, the Oklahoma Tobacco Helpline, and Florida’s Quit Your Way program. Each program offers free quitline services to their state’s residents. For each state, data were compared for 1 year prior to service changes to 1 year after services changed and promotions began. Registration and program utilization data from 21,918 (Minnesota); 64,584 (Oklahoma); and 141,209 (Florida) program enrollees were analyzed. Additionally, outcome study data from 1542 (Minnesota); 3377 (Oklahoma); and 3444 (Florida) program enrollees were analyzed. We examined treatment reach, satisfaction, 24-h quit attempts, 30-day point prevalence abstinence rates, select demographic characteristics, registration mode (post period only), and estimated number of quitters. Data were analyzed using χ^2^ analyses and t-tests.

**Results:**

Treatment reach rates increased by 50.62% in Oklahoma, 66.88% in Florida, and 480.56% in Minnesota. Significant increases in the estimated number of quitters were seen, ranging from + 42.75% to + 435.90%. Statistically significant changes in other variables (satisfaction, 24-h quit attempts, 30-day point prevalence abstinence rates, gender, and race) varied by state. During the post period, participants’ method of registration differed. Online enrollment percentages ranged from 19.44% (Oklahoma), to 54.34% (Florida), to 70.80% (Minnesota). In Oklahoma, 71.63% of participants enrolled by phone, while 40.71% of Florida participants and 26.98% of Minnesota participants enrolled by phone. Fax or electronic referrals comprised 8.92% (Oklahoma), 4.95% (Florida), and 2.22% (Minnesota) of program enrollees, respectively.

**Conclusions:**

Changing quitline services and implementing a new media campaign increased treatment reach and the estimated number of participants who quit smoking in three states. Quitline funders and tobacco control program managers may wish to consider approaches such as these to increase quitline utilization and population health impact.

## Background

Since the seminal article demonstrating quitline effectiveness was published in 2002 [1], this method of tobacco dependence treatment has become increasingly available. The World Health Organization reports that at least 53 countries provide national quitline services [2]. In the U.S., every state, the District of Columbia, Guam, and Puerto Rico have quitlines [3].

Despite the preponderance of meta-analyses demonstrating quitline effectiveness [4, 5], reaching tobacco users is a persistent challenge for quitlines. Across all U.S. quitlines, average treatment reach, defined as the relative proportion of the population of adult tobacco users that receives evidence-based treatment (telephone counseling and/or nicotine replacement therapy (NRT)) from a quitline, has ranged from a high of 1.19% in fiscal year 2009 to a low of .87% in fiscal year 2017 [6].

Declines in treatment reach represent an opportunity for innovation in the quitline space. Over the years, multiple studies have examined ways to increase quitline reach and utilization. Media campaigns are an effective tactic for promoting tobacco cessation and calls to quitlines [7–12]. Informed by the evidence base for tobacco cessation interventions, other strategies have focused on treatment modifications to increase reach: providing NRT without requiring enrollment in telephone counseling [13]; adding text messaging programs [14]; offering online cessation programs [15]; and including email support programs [16]. However, these innovations have rarely been widely implemented and promoted.

ClearWay Minnesota^SM^, an independent nonprofit organization funded with 3 % of Minnesota’s tobacco settlement, funds and administers QUITPLAN® Services (Minnesota’s free quitline). ClearWay Minnesota observed declines in use of its telephone counseling and online cessation programs over time, despite consistent promotion of these services. In March 2014, ClearWay Minnesota implemented a significant redesign of QUITPLAN® Services, with a goal of engaging more Minnesota tobacco users in the quitting process by offering more service options. Reviews of the literature, key informant interviews with experts in tobacco cessation, and formative research with smokers informed the program design [17]. The choices include the quitline (telephone counseling and NRT for uninsured and underinsured adult Minnesotans), as well as a set of treatment options that do not require enrollment in telephone counseling (i.e., one or more of the following: a 2 week “starter kit” of NRT, a stand-alone text messaging program, a stand-alone email program, and a printed quit guide for all adult Minnesotans) (see Table 1). In addition to expanded program offerings, the registration process was changed to allow online and telephone registration for all services. A mass media campaign, *“No Judgments. Just Help.”,* was implemented in April 2014 to promote the new services. ClearWay Minnesota evaluated QUITPLAN Services and found significant increases in the number of people enrolling in services and robust quit outcomes [18].

**Table 1:** State and State Tobacco Cessation Program Summary Descriptions

	Minnesota	Oklahoma	Florida
Population (2017)^a^	5,568,155	3,932,640	20,976,812
Smoking prevalence (2017)^b^	14.5%	20.1%	16.1%
Smokeless tobacco use prevalence (2017)^b^	4.8%	7.4%	2.7%
State tobacco cessation program summary description	QUITPLAN® Helpline – up to five telephone counseling calls, four weeks of nicotine replacement therapy (NRT) (combination NRT option), integrated text and email messaging, print materials. Individual QUITPLAN® Services – choice of two week “starter kit” of NRT (monotherapy only), standalone text messaging program, standalone email program, and/or printed quit guide. quitplan.com – unlimited access to self-directed website offered during the pre period only. (No NRT provided) (Program ended February 2014)	Oklahoma Tobacco Helpline- All Access: All callers receive one coaching call, at least 2 weeks of NRT (patches, gum or lozenges), print materials, integrated text, web coach. Uninsured, Medicare, IHS participants are eligible for 8 weeks of combination NRT (patches, gum or lozenges) and 5 calls with a quit coach. Web Coach – unlimited access to self-directed website, receive 2 weeks of NRT, emails, text, printed materials. Individual Services – choice of two week “starter kit” of NRT (monotherapy only), standalone text messaging program, standalone email program, and/or printed quit guide.	Quit Your Way – Phone Quit: up to three telephone counseling calls, two weeks of NRT (combination NRT option), integrated text and email messaging, integrated web coach messaging, printed quit guide. Web Quit: unlimited access to self-directed website, two weeks of NRT (combination NRT option), optional text and email messaging. Individual Quit Your Way services - choice of two week “starter kit” of NRT (monotherapy only), standalone text messaging program, standalone email program and/or printed quit guide.
Program eligibility criteria	QUITPLAN Helpline:– uninsured and underinsured^c^ Minnesotans age 13 years and older. -must be 18 years or older to receive NRT and text messaging. -can enroll twice in a 12-month period. Individual QUITPLAN Services: – all Minnesotans age 13 years and older. -must be 18 years or older to receive NRT and text messaging. -can receive two NRT “starter kits”, and all other services once, in a 12-month period.	All Oklahomans age 13 years and older are eligible for some level of services. NRT is provided only to those 18 years and older. Cost sharing partnerships with Medicaid and other private partners increase the number of calls and/or other services available to members. Oklahomans can enroll twice in all services in a 12-month period.	Services are open to all Floridians. NRT is provided only to those 18 years and older; other services available to those 13 years or older. Floridians can enroll twice in all services in a 12-month period.

^a^U.S. Census Bureau, https://www.census.gov/data/datasets/time-series/demo/popest/2010s-state-total.html

^b^Behavioral Risk Factor Surveillance System, https://www.cdc.gov/brfss/brfssprevalence/

^c^Self-reported lack of insurance coverage for telephone counseling and/or nicotine replacement therapy

Although Minnesota experienced success, it is unclear whether this approach to providing and promoting quitline services (i.e., offering service choices, ensuring tobacco users could register for all services either online or by telephone, and promoting services using the *“No Judgments. Just Help.*” media campaign) and its resultant positive outcomes could be replicated in other states. Oklahoma and Florida expressed interest in this approach. Both states were concerned about declining quitline call volumes and utilization, despite ongoing, robust media campaigns promoting their state quitlines. Additionally, both Oklahoma and Florida recognized the importance of improving how they used technology to better reach their state’s tobacco users. Oklahoma implemented changes to its services in 2015 and Florida in 2016. Both states also added online registration for all services and used the *“No Judgments. Just Help.”* campaign to promote the new services. All three states use the same quitline service provider (Optum®), which facilitated consistent implementation of these changes.

In this paper, we report quitline registration and evaluation data from three states: Minnesota, Oklahoma, and Florida, before and after each state made changes to their service offerings and promotional campaigns. The goal of this analysis was to understand whether the changes in enrollments and quit outcomes observed in Minnesota were also experienced in other states that differ from each other in multiple ways. Although Minnesota has previously reported some data on their experience after changing services, data examining pre-post differences in key outcomes were not analyzed or reported [18]. Moreover, pre-post comparisons from three states on metrics such as treatment reach, quit attempts, quit rates, and participant satisfaction have not been previously reported. Finally, we estimated the number of successful quitters in each state before and after changes were made to services and promotions to gauge the potential impact of these changes. These novel findings are relevant to other state tobacco control programs, quitline service providers and quitline funders seeking to improve program reach.

## Methods

### Design

This observational study reports findings from two analyses: (1) an analysis of quitline registration data and (2) an analysis of quitline evaluation data.

Data were collected from participants who enrolled in state quitline services operated by the states of Minnesota, Oklahoma, and Florida. Participants chose which service(s) they wished to enroll in, based on each state’s program eligibility criteria. Table 1 reports each states’ population, smoking and smokeless tobacco prevalence data, state-funded cessation programs offered to state residents, and program eligibility criteria.

Table 2 reports the time periods analyzed for each state. We defined the “pre” period as the 12 months prior to the month that services changed and the new promotional campaign began. Each state had a “soft launch” of their new services. Although state residents could enroll in the new services during the soft launch period, the states were conducting quality control activities to ensure that the services were being delivered as intended and that data were being collected accurately. The states also did not promote the new services during this period. Since the length of the soft launch period varied by state (Minnesota: 1 month; Oklahoma: 3 months; Florida: 7 months), this period was excluded from the analysis to ensure that the “post” period was comparable across states. We defined the “post” period as the 12 months after the soft launch period ended (i.e., the new services were available, quality control activities had been completed, and promotions had begun).

**Table 2:** Study Time Periods by State

State	Pre	Soft launch^a^	Post
Minnesota	Mar 2013 - Feb 2014	Mar 2014	Apr 2014 - Mar 2015
Oklahoma	Jul 2014 - Jun 2015	Jul 2015 - Sep 2015	Oct 2015 - Sep 2016
Florida	Jan 2015 - Dec 2015	Jan 2016 - Jul 2016	Aug 2016 - Jul 2017

^a^Soft launch: period when the new services were offered, but states were completing quality control activities and no media was promoting the services. The soft launch period for each state was excluded from analysis

The first analysis examined each quitlines’ program registration data collected by the quitline service provider to assess whether there were differences among participants across states. Data from all program registrants in the time periods studied (Table 2) were included in this analysis.

The second analysis compared outcome evaluation data collected by each state to gauge whether quit outcomes varied by state. In each state, a sample of eligible participants was invited to respond to a follow-up survey (phone or online) 7 months after they enrolled in the state’s quitline program. The follow-up surveys for all three states were conducted by the same evaluation firm (Professional Data Analysts); sample sizes and sampling method (exhaustive, random, or random stratified by program) were determined based on evaluation needs for each state. When samples were stratified by program, weights were applied to adjust responders back to population percentages. Survey protocols were similar in each state: pre-notification letters, up to 15 call attempts and 5 email reminders, and incentives ranging from a $2 bill to a $10 check. Outcome evaluation flowcharts for each state’s outcome studies – including sampling method – are provided in Table 3. Survey respondents within each state were weighted by sampling strata.

**Table 3:** Outcome Evaluation Flowcharts

	Minnesota Pre Period	Minnesota Post Period
Oct 2011-Dec 2011		May 2014-Jul 2014
Helpline Enrollments (*N* = 895)		Helpline and Individual Services Enrollments (*N* = 5031)
Excluded (*n* = 311) • Sampled for an unrelated evaluation study (*n* = 267) • Did not consent (*n* = 38) • Less than 18 years old (*n* = 2) • Duplicate enrollments (*n* = 4)		Excluded (*n* = 1429) • Non-tobacco user (*n* = 177) • Did not enroll (e.g., called for information only) (*n* = 441) • Prior enrollment in study period (*n* = 147) • Did not consent (*n* = 658) • Less than 18 years old (*n* = 1) • No contact information (*n* = 5)
Eligible for Follow-up (*N* = 584)		Eligible for Follow-up (*N* = 3602)
Exhaustively sampled (*N* = 584)		Randomly sampled within program strata: Helpline, Individual Services (IS) with NRT only, IS with NRT and text/email, IS with text/email only (*N* = 2006)
Responded to survey (*N* = 372)		Responded to survey (*N* = 1170)
Study participants *N* = 372		Study participants *N* = 1170
Excluded for quit rates (*n* = 54) • Did not receive phone counseling or NRT (*n* = 48) • Quit for 30 days or more at intake (*N* = 6)		Excluded for quit rates (*n* = 78) • Did not receive phone counseling or NRT (*n* = 73) • Quit for 30 days or more at intake (*n* = 5)
Study participants included in quit rate *N* = 318		Study participants included in quit rate *N* = 1092
Oklahoma Pre Period		Oklahoma Post Period
Jul 2014-Jun 2015	May 2013-Sep 2013	Oct 2015-Sep 2016
Helpline Enrollments (*N* = 24,882)	Web Coach Enrollments (*N* = 1903)	Helpline, Web Coach, and Individual Services Enrollments (*N* = 37,175)
Excluded (*n* = 9690) • Did not consent (*n* = 2520) • Less than 18 years old (*n* = 84) • Non-OK resident (*n* = 1) • Non-English speaker (*n* = 157) • Invalid contact information (*n* = 677) • Did not receive phone counseling or NRT (*n* = 3123) • Prior enrollment in past 12 months (*n* = 2336) • Sampled for survey in past 12 months (*n* = 792)	Excluded (*n* = 1100) • Did not consent (*n* = 324) • Less than 18 years old (*n* = 8) • Non-English speaker (*n* = 7) • Did not log into web program (*n* = 705) • Prior enrollment in past 12 months (*n* = 30) • Sampled for survey in past 12 months (*n* = 26)	Excluded (*n* = 13,851) • Did not consent (*n* = 3124) • Less than 18 years old (*n* = 109) • Non-OK resident (*n* = 2) • Non-English speaker (*n* = 207) • Invalid contact information (*n* = 195) • Did not receive phone counseling or NRT (*n* = 6318) • Prior enrollment in past 12 months (*n* = 2319) • Sampled for survey in past 12 months (*n* = 1577)
Eligible for Follow-up (*N* = 15,192)	Eligible for Follow-up (*N* = 803)	Eligible for Follow-up (*N* = 23,324)
Randomly sampled (*N* = 2475)	Randomly sampled (*N* = 709)	Randomly sampled (*N* = 3320)
Responded to survey (*N* = 1273)	Responded to survey (*N* = 356)	Responded to survey (*N* = 1748)
Study participants *N* = 1273	Study participants *N* = 356	Study participants *N* = 1748
Excluded for quit rates (*n* = 7) • Quit for 30 days or more at intake (*n* = 7)	Excluded for quit rates (*n* = 75) • Did not receive phone counseling or NRT (*n* = 74) • Quit for 30 days or more at intake (*n* = 1)	Excluded for quit rates (n = 5) • Quit for 30 days or more at intake (n = 5)
Study participants included in quit rate *N* = 1266	Study participants included in quit rate *N* = 281	Study participants included in quit rate *N* = 1743
Florida Pre Period		Florida Post Period
Jan 2015-Dec 2015		Aug 2016-Jul 2017
Helpline and Web Coach Enrollments (N = 58,213)		Helpline, Web Coach and Individual Services Enrollments (*N* = 103,709)
Excluded (*n* = 5787) • Did not consent (*n* = 5564) • Less than 18 years old *(n* = 73) • Non-FL resident (*n* = 3) • Sampled for same survey within past 4 months (*n* = 147)		Excluded (*n* = 41,320) • Prior enrollment in past 12 months (*n* = 23,226) • Did not consent (*n* = 16,407) • Quit for past 30 days at intake (*n* = 392) • No contact information (*n* = 109) • Less than 18 years old (*n* = 44) • Non-FL resident (*n* = 70) • Sampled for same survey within past 4 months (*n* = 602) • Individual Services enrollment with no service received (*n* = 470)
Eligible for Follow-up (*N* = 52,426)		Eligible for Follow-up (*N* = 62,389)
Randomly sampled within program strata: Helpline and Web Coach (*N* = 3151)		Randomly sampled (*N* = 3519)
Responded to survey (*N* = 1676)		Responded to survey (*N* = 1768)
Study participants *N* = 1676		Study participants *N* = 1768
Excluded for quit rates (*n* = 459) • Did not receive phone counseling or NRT (*n* = 383) • Quit for 30 days or more at intake (*n* = 76)		Excluded for quit rates (*n* = 196) • Did not receive phone counseling or NRT (*n* = 196)
Study participants included in quit rate *N* = 1217		Study participants included in quit rate *N* = 1572

### Sample

Quitline registration data were examined from 21,918 (Minnesota), 64,584 (Oklahoma), and 141,209 (Florida) program registrants. The number of program registrants included in each state’s outcome study varied (see Table 3).

### Measures

Variables analyzed in the evaluation of registration data were registration mode (phone, online, or fax/electronic referral) and select demographic (self-reported gender, age, and race) measures. Registration mode data are reported only for the post period, since the participating states did not offer phone, online, and fax/electronic referral enrollment options for all services prior to implementing changes. Race is not reported for Minnesota due to significant variation in how these data were collected over time, making pre-post comparisons unreliable. Ethnicity is not reported due to incomplete data across states and programs.

Data from outcome evaluations were analyzed to measure changes in satisfaction, quit attempt rates, and 30-day point prevalence abstinence rates. All outcomes were calculated among survey responders. Quit attempts (“Since you [enrolled/registered for services] about seven months ago, did you stop using tobacco for 24 hours or longer because you were trying to quit?”); 30-day point prevalence abstinence (“Have you smoked any cigarettes or used other tobacco, even a puff or pinch, in the last 30 days?”); and satisfaction (“Overall, how satisfied were you with the service you received from the quitline?”) were assessed using the North American Quitline Consortium (NAQC) Minimal Data Set questions [19]. While the core outcome study evaluation measures did not vary, each state customized the question by adding its program name. The NAQC guidelines for calculating 30-day point prevalence abstinence rates were followed [20]. The four satisfaction responses were dichotomized into “Very satisfied”/“Mostly satisfied” and “Somewhat satisfied”/“Not at all satisfied” to reduce comparisons.

Both Census population estimates and Behavioral Risk Factor Surveillance System smoking/smokeless prevalence data were used in treatment reach analyses. Treatment reach is a standard metric for measuring relative quitline reach used by NAQC and is defined as the number in the target population who received evidence-based treatment (telephone counseling calls/and or NRT) from a quitline divided by the total target population [21].

The impact of changing services in each state was estimated by multiplying the 30-day point prevalence abstinence rate by the number of participants who received telephone counseling calls and/or NRT. Other services were excluded from this analysis to align with NAQC’s definition of evidence-based treatment.

### Analysis

Data were analyzed in 2018. Pre-post differences in participant demographic characteristics, quit attempts, and 30-day point prevalence abstinence within each state were compared using chi-square tests for categorical measures and t-tests for age. Histograms were used to visually check for normality in age distributions before conducting t-tests. All analysis was conducted using SAS 9.4.

## Results

Table 4 reports numbers of enrollees, treatment reach, quit attempts, and 30-day point prevalence abstinence rates observed in each state before and after changes were made to services. All states saw substantial increases in the numbers of tobacco users enrolling in their programs. Minnesota observed more than a fivefold increase in treatment reach (.36% pre vs. 2.09% post; 480.56% increase) after changes were made to services; Oklahoma and Florida reported increases in their state’s treatment reach of 50.62% (3.22% pre vs. 4.85% post) and 66.88% (1.57% pre vs. 2.62% post), respectively.

**Table 4:** Pre-Post Numbers Enrolled, Evidence-Based Treatment Received, Treatment Reach, Quit Attempts, Abstinence Rates, and Estimated Quitters

	Pre	Post	
Number enrolled	N	N	
Minnesota	5599	16,319	
Oklahoma	28,950	35,634	
Florida	55,242	85,967	
Number of unique tobacco users receiving evidence-based treatment^a^	N	N	
Minnesota	2784	14,880	
Oklahoma	22,241	29,763	
Florida	41,982	74,039	
Treatment reach	% (95% CI)	% (95% CI)	% change
Minnesota	0.36 (0.34–0.38)	2.09 (2.00–2.18)	480.56
Oklahoma	3.22 (3.00–3.47)	4.85 (4.52–5.23)	50.62
Florida	1.57 (1.47–1.69)	2.62 (2.45–2.83)	66.88
			Significance Test
24-h quit attempt rate	% (95% CI)	% (95% CI)	*Χ*^*2*^ (*p*-value)
Minnesota	89.94 (85.92–92.95)	84.13 (81.83–86.19)	6.26 (0.01*)
Oklahoma	87.04 (85.26–88.64)	86.98 (85.28–88.50)	2.37 (0.12)
Florida	84.96 (82.89–86.90)	85.08 (83.18–86.80)	0.00 (1.00)
30-day point prevalence abstinence rate	% (95% CI)	% (95% CI)	*Χ*^*2*^ (*p-*value)
Minnesota	26.10 (21.43–31.37)	26.18 (23.65–28.87)	0.00 (1.00)
Oklahoma	29.69 (27.46–32.02)	31.67 (29.50–33.92)	1.44 (0.23)
Florida	35.50 (32.86–38.23)	30.28 (28.02–32.65)	8.21 (0.004**)
Estimated number quit^b^			% change
Minnesota	727	3896	435.90%
Oklahoma	6603	9426	42.75%
Florida	14,904	22,419	50.42%

Boldface indicates statistical significance (*p < 0.05, **p < .01)

^a^Evidence-based treatment is defined as receiving telephone counseling calls and/or nicotine replacement therapy

^b^Estimated number quit is calculated by multiplying the number of unique tobacco users receiving evidence-based treatment by the 30-day point prevalence abstinence rate

More than 80% of participants in each state reported making a 24-h quit attempt during both the pre and post periods. Minnesota saw a statistically significant reduction in 24-h quit attempts in the post period compared to the pre period (89.9% vs. 84.1%, *p* = .01); no change was observed in Oklahoma and Florida. Thirty-day point prevalence abstinence rates increased in Oklahoma, but the increase was not statistically significant. No change was seen in Minnesota. Florida observed a statistically significant decline in abstinence rates (35.50% vs. 30.28%, *p* = .004). Graphs of point estimates and 95% confidence intervals for study outcomes can be found in Additional file 1: Digital Content 1.

The impact of changing services on the number of people who received evidence-based treatment from each state’s program and who successfully quit in 1 year’s time was estimated (Table 4). The number of estimated quitters in each state increased, although the rate of increase varied (Minnesota, 435.90% increase; Oklahoma, 42.75% increase; Florida, 50.42% increase).

Table 5 summarizes changes in gender, race, and mean age in the pre and post periods. All states saw statistically significant changes in the percentage of men enrolling in their programs. The largest increase was seen in Minnesota; Florida saw a slight increase and Oklahoma saw a slight decrease. No significant differences in race were seen in Oklahoma; Florida saw a significant (*p* < .0001) change, with the largest increase in those reporting White race and the largest decrease in those reporting Black or African American race. All states saw statistically significant changes in average age of participants. Both Minnesota and Florida saw slight decreases in average age, and Oklahoma saw a slight increase in average age.

**Table 5:** Pre-Post Comparisons of Gender, Race, and Age

	Pre		Post		*Χ*^2^	*p*-value
	N	%	N	%		
Gender						
Minnesota					76.32	<.0001**
Female	3458	61.76%	8979	55.05%		
Male	2141	38.24%	7333	44.95%		
Oklahoma					5.90	.02*
Female	16,417	56.74%	20,548	57.69%		
Male	12,519	43.26%	15,070	42.31%		
Florida					15.48	<.0001**
Female	31,892	57.75%	48,718	56.69%		
Male	23,331	42.25%	37,223	43.31%		
Race						
Minnesota	^a^		^a^			
Oklahoma					4.64	.2003
White	21,294	75.32%	25,605	75.42%		
Black or African American	2633	9.31%	3020	8.89%		
American Indian	3186	11.27%	3944	11.62%		
Other	1159	4.10%	1383	4.07%		
Florida					347.24	<.0001**
White	40,514	77.87%	67,187	81.97%		
Black or African American	6357	12.22%	7892	9.63%		
American Indian	673	1.29%	905	1.10%		
Other	4482	8.61%	5985	7.30%		
Age (mean)	Years		Years		t	*p-*value
Minnesota	43.14		41.85		5.94	<.0001**
Oklahoma	43.83		44.60		−6.59	<.0001**
Florida	46.30		45.88		5.44	<.0001**

Boldface indicates statistical significance (*p < 0.05, **p < 0.0001)

^a^Race not reported for Minnesota due to significant variation in how these data were collected over time, making pre-post comparisons unreliable

Some changes were observed in satisfaction with services in the pre and post periods (see Additional file 1: Digital Content 1 and Additional file 2: Digital Content 2). No significant changes in satisfaction rates were observed in Minnesota. A significant increase in satisfaction rates was seen in Oklahoma, and a significant decrease in satisfaction rates was seen in Florida. Across all states in both time periods, at least 74.64% of participants reported being very or mostly satisfied with services received.

During the post period, the ways in which participants enrolled in each state’s programs varied. In Minnesota, 70.80% of participants enrolled online, 26.98% enrolled by phone, and 2.22% enrolled via either fax referral or electronic referral. In Oklahoma, 19.44% of participants enrolled online, 71.63% enrolled by phone, and 8.92% enrolled via either fax referral or electronic referral. In Florida, 54.34% of participants enrolled online, 40.71% enrolled by phone, and 4.95% enrolled via either fax or electronic referral.

## Discussion

Adding cessation service options to existing quitline services and promoting the new service options resulted in substantial increases in the number of tobacco users that enrolled in each state’s cessation services, as well as the number that were estimated to have successfully quit. While the magnitude of the increases varied by state, these results suggest that such changes have the potential to boost quitting at the population level and yield a substantial positive public health impact. If each state had not changed services, it may have taken Minnesota over 5 years, Florida 1.5 years, and Oklahoma 1.4 years, to help the same number of tobacco users successfully quit.

These findings also demonstrate that these changes can still result in a positive public health impact, even if overall quit rates fall. In this analysis, Florida saw a reduction in its overall quit rate after implementing these changes; however, the estimated number of quitters still increased by over 50% due to the large increase in the number of tobacco users receiving services. Potential explanations of the change in quit rates include, but are not limited to, the changes in services and promotional campaign; or demographic or clinical differences in outcome study participants over time. Regardless of the reason(s) for this significant change in quit rates, Florida’s quit rate remained robust and is above the NAQC standard of 30% [6]; satisfaction with services also remained high.

These findings also represent a possible strategy to increase quitline treatment reach. Each state saw substantial increases in utilization of evidence-based services provided by their state quitline, although the magnitude of these increases varied across states. Increasing tobacco users’ use of evidence-based cessation services is one of the keys to reducing tobacco’s harm. Each year in the U.S. alone, tobacco use results in more than 480,000 premature deaths and in $300 billion in excess expenditures due to smoking-related illnesses [8, 22]. Studies show that delivering tobacco use screening and brief cessation interventions to adults, and providing tobacco use screening and counseling to youth, are two of the top three most clinically-effective and cost–effective clinical preventive services [23]. Moreover, the positive health effects of smoking cessation are well documented [8]. Implementing tactics to increase quitline treatment reach such as those reported in this paper have the potential to increase quitline’s public health impact.

These findings may also help increase quitline treatment reach by engaging tobacco users without requiring them to speak to a coach. Previous research has found that while some tobacco users are eager to use quitline services, others perceive calling a quitline to be inconsistent with their desire to quit on their own, even though this desire does not prevent them from considering using support such as NRT or text messaging [17]. Providing access to services using an online registration process allowed tobacco users to register and receive the services they desired without ever having to speak to a quitline counselor. In order to increase quitline reach and achieve NAQC’s goal of reaching 6% of tobacco users [6], these data suggest that states not only need to promote services but consider adding service options and online enrollment to engage more tobacco users in the quitting process.

Some differences in 24-h quit attempt rates, 30-day point prevalence abstinence rates and participant satisfaction were observed; however, no consistent pattern emerged across states. It is possible that some participants were motivated to try to quit due to each state’s change in services and new promotional campaign, but did not succeed, which may have affected their satisfaction with services. Research has demonstrated that providing cessation support to smokers, regardless of their motivation to quit, increases quit attempts and other positive changes in smoking behavior; the impact on abstinence is mixed [24–26]. Importantly, all of the quit rates in this evaluation are robust. The United States Public Health Service Clinical Practice Guideline reports a range of quit rates for quitlines offering both telephone counseling and NRT of 24.5% to 32%; all of the quit rates reported in this evaluation are within that range [4]. Moreover, both Oklahoma and Florida’s quit rates exceed NAQC’s 30-day point prevalence abstinence rate goal of 30% for state quitlines [6].

Each of the states in this analysis differs in many ways, including but not limited to total population, demographics such as age, race and ethnicity, median income, smoking and smokeless tobacco use rates, and percentage of the state’s population living in urban and rural areas [27]. Each state also offers different service options and has different program eligibility criteria (Table 1). All of these factors may have contributed to the differences in participant characteristics, satisfaction and quit outcomes examined in this study. For example, online enrollment may have greater appeal to younger tobacco users. Minnesota and Florida saw much higher rates of online enrollment compared to Oklahoma, which may have affected the mean age of participants in each state’s services. Both the addition of a two-week “starter kit” of NRT that could be ordered either online or by phone, as well as some changes to program eligibility criteria, likely contributed to the variation observed across states. Prior to changing services, NRT was only available to uninsured and underinsured Minnesotans who enrolled in telephone counseling, while Oklahoma and Florida offered NRT to all adult residents of their state through both their online cessation program and their telephone counseling program. Minnesota expanded access to NRT when the new services were implemented, both by adding a NRT “starter kit” that was available to all adult Minnesotans, as well as allowing Minnesotans to order NRT “starter kits” online and by phone. This may have explained the greater increase in program enrollments as well as treatment reach in Minnesota compared to Oklahoma and Florida.

There are several limitations to this analysis. The data reported in this study are from observational cross-sectional program evaluations conducted at different time points, so we cannot discount the possibility of secular trends influencing these findings. However, each state’s increases were reported after noticing declining utilization of quitline services (data not shown), and NAQC has reported reductions in quitline calls and treatment reach [6]. The impact factor analysis used NAQC’s responder quit rate, which includes only those who received telephone counseling and/or NRT. Both use of this quit rate (rather than a more conservative measure such as intention to treat) as well as exclusion of other services offered by the participating states that may affect quit outcomes would affect the results of this analysis. However, using the responder quit rate and excluding other services is consistent with NAQC’s recommendations [20]. We were also limited in the variables we could analyze across states due to differences in data collected by each state’s program and how questions were asked over time, since state quitlines tailor data collected from program participants to meet their state’s unique needs.

## Conclusions

This study demonstrates that three states, each with very different demographic characteristics and existing quitline program offerings, were able to significantly increase treatment reach after changing service offerings, adding online registration for all services, and promoting the new service options with a fresh campaign. Differences in each state’s experience can likely be attributed to the states’ prior program offerings and unique context. These findings suggest that if states are interested in increasing quitline reach, approaches such as these should be considered to engage more tobacco users in the quitting process and increase the positive population health impact of their programs.

## Supplementary information

**Additional file 1.** Point estimates and 95% confidence intervals for 24-hour quit attempts, 30-day point prevalence abstinence, and satisfaction (very or mostly satisfied)

**Additional file 2.** Changes in Satisfaction with Services Received

## Data Availability

The datasets used and/or analysed during the current study are available from the corresponding author on reasonable request.

## Abbreviations

NAQC: North American Quitline Consortium
NRT: Nicotine replacement therapy
