# Supplementary material for: Replicating state Quitline innovations to increase reach: findings from three states
Source: BMC Public Health. 2020 Jan 6;20:7. doi: 10.1186/s12889-019-8104-3 (PMC6945575; doi:10.1186/s12889-019-8104-3)
Supplement: Supplementary file 1 — Additional file 1. Point estimates and 95% confidence intervals for 24-hour quit attempts, 30-day point prevalence abstinence, and satisfaction (very or mostly satisfied) [file 12889_2019_8104_MOESM1_ESM.docx]

Additional file 1: Point estimates and 95% confidence intervals for 24-hour quit attempts, 30-day point prevalence abstinence, and satisfaction (very or mostly satisfied)
