# Supplementary material for: Replicating state Quitline innovations to increase reach: findings from three states
Source: BMC Public Health. 2020 Jan 6;20:7. doi: 10.1186/s12889-019-8104-3 (PMC6945575; doi:10.1186/s12889-019-8104-3)
Supplement: Supplementary file 2 — Additional file 2. Changes in Satisfaction with Services Received [file 12889_2019_8104_MOESM2_ESM.docx]

Additional file 2: Changes in Satisfaction with Services Received

|  | Pre | | Post | |  |  |
| --- | --- | --- | --- | --- | --- | --- |
| State | Very or mostly satisfied | Somewhat or not at all satisfied | Very or mostly satisfied | Somewhat or not at all satisfied | *Χ*^2^ | p-value |
| Minnesota | 78.05% | 22.95% | 74.64% | 25.36% | 1.58 | 0.21 |
| Oklahoma | 75.79% | 24.21% | 80.97% | 19.03% | 12.88 | **<0.001** |
| Florida | 84.20% | 15.80% | 75.86% | 24.14% | 29.66 | **<0.001** |

Boldface indicates statistical significance (p<0.001).
